# Overcoming Decisional Gaps in High-Risk Prescribing by Junior Physicians Using Simulation-Based Training: Protocol for a Randomized Controlled Trial

**DOI:** 10.2196/31464

**Published:** 2022-04-27

**Authors:** Julie C Lauffenburger, Matthew F DiFrancesco, Renee A Barlev, Ted Robertson, Erin Kim, Maxwell D Coll, Nancy Haff, Constance P Fontanet, Kaitlin Hanken, Rebecca Oran, Jerry Avorn, Niteesh K Choudhry

**Affiliations:** 1 Brigham and Women's Hospital, Harvard Medical School Boston, MA United States; 2 ideas42 New York, NY United States

**Keywords:** pragmatic trial, behavioral science, prescribing, benzodiazepines, antipsychotics, impact evaluation

## Abstract

**Background:**

Gaps between rational thought and actual decisions are increasingly recognized as a reason why people make suboptimal choices in states of heightened emotion, such as stress. These observations may help explain why high-risk medications continue to be prescribed to acutely ill hospitalized older adults despite widely accepted recommendations against these practices. Role playing and other efforts, such as simulation training, have demonstrated benefits to help people avoid decisional gaps but have not been tested to reduce overprescribing of high-risk medications.

**Objective:**

This study aims to evaluate the impact of a simulation-based training program designed to address decisional gaps on prescribing of high-risk medications compared with control.

**Methods:**

In this 2-arm pragmatic trial, we are randomizing at least 36 first-year medical resident physicians (ie, interns) who provide care on inpatient general medicine services at a large academic medical center to either intervention (simulation-based training) or control (online educational training). The intervention comprises a 40-minute immersive individual simulation training consisting of a reality-based patient care scenario in a simulated environment at the beginning of their inpatient service rotation. The simulation focuses on 3 types of high-risk medications, including benzodiazepines, antipsychotics, and sedative hypnotics (Z-drugs), in older adults, and is specifically designed to help the physicians identify their reactions and prescribing decisions in stressful situations that are common in the inpatient setting. The simulation scenario is followed by a semistructured debriefing with an expert facilitator. The trial’s primary outcome is the number of medication doses for any of the high-risk medications prescribed by the interns to patients aged 65 years or older who were not taking one of the medications upon admission. Secondary outcomes include prescribing by all providers on the care team, being discharged on 1 of the medications, and prescribing of related medications (eg, melatonin, trazodone), or the medications of interest for the control intervention. These outcomes will be measured using electronic health record data.

**Results:**

Recruitment of interns began on March 29, 2021. Recruitment for the trial ended in Q42021, with follow-up completed by Q12022.

**Conclusions:**

This trial will evaluate the impact of a simulation-based training program designed using behavioral science principles on prescribing of high-risk medications by junior physicians. If the intervention is shown to be effective, this approach could potentially be reproducible by others and for a broader set of behaviors.

**Trial Registration:**

ClinicalTrials.gov NCT04668248; https://clinicaltrials.gov/ct2/show/NCT04668248

**International Registered Report Identifier (IRRID):**

PRR1-10.2196/31464

## Introduction

Decisional gaps between how individuals behave when in an emotional or “hot” state (sometimes called “System 1” thinking) in contrast to when they are better able to consider issues more rationally (ie, “System 2” thinking) are increasingly being recognized as a reason why people make suboptimal decisions in stressful situations [1-6]. For example, the “hot-cold empathy gap” helps to explain the observation that when people are in rational or “cold” states, they incorrectly predict what their behavior will be during “hot states” [5]. Other related behavioral principles describe deliberative versus impulsive thinking and have found similar gaps in behaviors when people are in different states [7-9]. Moreover, often in these “hot states,” there may be limited time to make decisions leading to mental shortcuts, often called “heuristics,” that could lead to suboptimal decision making [10].

These decisional gaps are believed to be a central reason why health care providers underestimate the likelihood that they will make suboptimal prescribing decisions in stressful situations [11]. For example, the use of medications such as antipsychotics, benzodiazepines, and sedative hypnotic “Z-drugs,” to manage delirium and agitation for hospitalized patients remains highly prevalent despite considerable risks associated with their use and guidelines that recommend their avoidance in older adults [12-15]. In this context, there are numerous factors that are stressful for physicians which promote System 1 thinking. These include clinical complexity, perceived pressure from nursing staff, patients and their caregivers, and fatigue [11,16,17]. These issues may be particularly challenging for junior physicians, especially medical residents, given their relative lack of experience. Further, interventions to reduce the prescribing of these high-risk medications have typically focused on educating providers by transmitting facts alone, and have only been modestly successful, perhaps, because they have underestimated the importance of stress faced by clinicians in the real-life care of complex and acutely ill inpatients [1,18-22].

Efforts to address decisional gaps between System 1 and System 2 thinking in other fields have involved role-playing or other games to simulate System 2 thinking states, such as having participants evaluate their cravings for tobacco during hot state and cold state sessions [1,2,23,24]. In clinical medicine, simulation has increasingly been used to help health care professionals, alone and in teams, practice how they would handle stressful situations such as cardiac arrest or emergent trauma situations in emergency rooms [25-34]. By extension, these approaches could help address decisional gaps for prescribing high-risk medications for older adults.

Accordingly, we launched a pilot trial to evaluate the impact of a simulation-based training program designed to address decisional gaps between System 1– and System 2–driven choices compared with online educational training on high-risk prescribing by first-year medical residents (ie, interns) at a large academic medical center. We hypothesized that the simulation-based training program would reduce high-risk prescribing by interns compared with online educational training.

## Methods

### Overall Study Design

**Figure 1:**
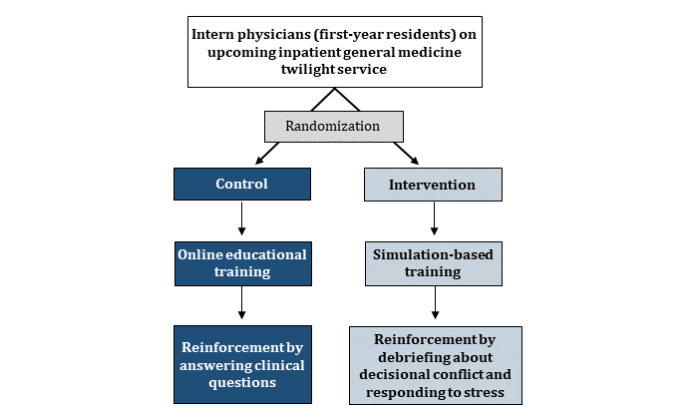
Overall trial design.

We designed and launched a 2-arm pragmatic randomized trial to evaluate the impact of a simulation-based training program on the prescribing of high-risk medications to hospitalized older adults (Figure 1). The specific medication classes of interest are benzodiazepines, antipsychotics, and sedative hypnotics (Z-drugs), the use of which is strongly discouraged by major clinical guidelines [15,35].

The authors will be responsible for performing the study analyses, writing the first draft of the manuscript, substantive edits, and submitting its final contents for publication. Data analysts at the end of the study will be blinded to arm assignment; residents are not blinded due to the nature of the interventions and need to provide informed consent for participation.

### Ethical Approval

The trial is approved by the Institutional Review Board of Brigham and Women’s Hospital (BWH; Mass General Brigham) approval number 2020P003643, and registered with ClinicalTrials.gov (NCT04668248).

### Study Setting and Participants

The study is being conducted at the BWH Main and Faulkner campuses, an academic medical center in Boston, Massachusetts, affiliated with Harvard Medical School. Potentially eligible participants are physician interns (ie, first-year medical resident physicians) practicing on the general medicine inpatient services at BWH assigned to an evening rotation. At BWH Main campus, these services include the General Medicine Service or Integrated Teaching Unit; at BWH Faulkner, this service includes a General Medicine Service. These services consist of 8 teams of residents and interns, rotating on daytime and evening shifts.

We chose to focus on interns starting on an evening rotation as they cover the shifts during which the high-risk medications of interest are most often prescribed. In addition to being the least experienced physicians on the medical team, they are also the first and primary point of contact for nurses, pharmacists, and other specialists. Given the busy pace of these evening rotations [36,37], the interns may also be prone to make decisions using System 1 thinking, which was supported by interviews we conducted (see the section “Intervention: Simulation Training” for more detail).

Older adults (≥65 years) admitted to 1 of these services not previously taking 1 of the high-risk medications of interest upon admission will be the target population for analyses.

### Study Procedures and Randomization

**Figure 2:**
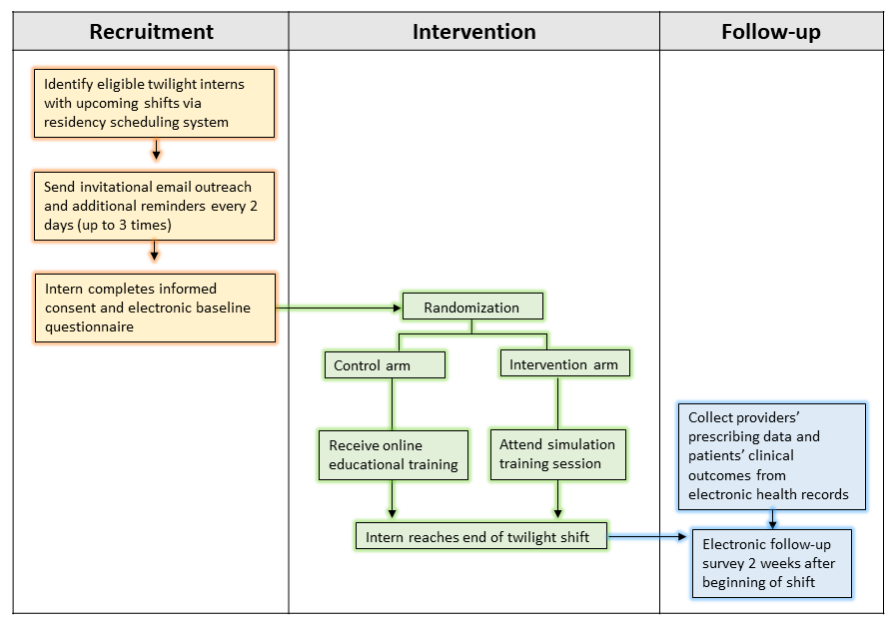
Timeline of study procedures.

The timeline of study procedures is shown in Figure 2. Potentially eligible interns were invited by email to join a study using educational training to reduce prescribing of “high-risk medications.” No information was provided in advance about the exact medication classes to avoid any potential bias in the educational trainings.

Interns interested in participating were asked to provide written informed consent and complete a baseline questionnaire administered and collected through the REDCap electronic data capture tool housed at BWH prior to randomization. REDCap is a secure web-based software platform supporting data collection for research studies and is housed on the BWH server [38,39]. The baseline assessment includes questions about demographic information, specifically sex, age, race, and ethnicity; the 6-item State-Trait Anxiety Inventory (STAI-6) questions [40,41], which measure types of anxiety and we used these questions to query about stressful prescribing decisions specifically (Multimedia Appendix 1); and the 8-item Revised Physicians’ Reactions to Uncertainty Scales, which ask about anxiety due to uncertainty and concern for bad outcomes (Multimedia Appendix 2) [42].

Interns who consent were randomized in a 1:1 ratio to 1 of 2 arms: (1) Arm 1—simulation training; and (2) Arm 2—control (online educational training) using a simple random number generator within REDCap. To improve baseline participant balance between the 2 treatment arms, we used stratified block randomization based on their service (ie, General Medicine Service, Integrated Teaching Unit, or Faulkner). Each potentially eligible intern will only participate in the trial once. Both intervention and control trainings were designed to be completed on the same day of the interns’ evening rotation (eg, the second day of their rotation). They were also similar in duration, both designed to take about 40 minutes altogether.

Interns in both arms were asked to complete a follow-up survey 2 weeks after they begin their eligible evening rotation. This follow-up questionnaire repeats the STAI-6, the Revised Physicians’ Reactions to Uncertainty Scales, and asks about satisfaction with the clarity and relevance of the information provided, timing of information, extent of knowledge gained, and expectations for using this information moving forward [40-42]. As described in the informed consent documentation, providers in both arms received US $25 for completing the baseline survey, US $75 for completing the training (either intervention or control as applicable), and US $50 after completing the follow-up questionnaire.

### Intervention: Simulation Training

#### Design Process

Prior to designing the simulation-based training program, we conducted in-depth qualitative interviews with 25 medical residents and allied health professionals to understand barriers to reducing prescribing of high-risk medications in stressful situations. To avoid potential contamination with the trial, the medical trainees were based at a different academic medical center in Boston (Massachusetts General Hospital) or had recently finished their medicine internship and were currently pursuing residency training in other noninternal medicine programs (ie, neurology or dermatology). These interviews clarified the contributors to decisional conflict and factors associated with high-risk prescribing and stress, including most notably time pressures, perceived pressure from nurses, and stress due to lack of familiarity with new patients (data shown elsewhere). The interns also clarified a general lack of didactic training on prescribing of high-risk medications.

#### Simulation-Based Intervention

The simulation intervention consists of a 40-minute immersive simulation conducted at the Neil and Elise Wallace STRATUS Center for Medical Simulation at BWH. The intervention includes the scenario itself and a debriefing session. Each session is led and moderated by an expert physician facilitator and staffed by 2 different clinically trained “actors” (ie, practicing nurses) in simulated hospital rooms.

Based upon the qualitative interviews, the simulation-based training was designed to help interns identify their reactions and prescribing decisions common in the inpatient setting when they are in the “hot state” triaging multiple demands for their attention. The scenarios require the participants to simultaneously care for several patients through in-person, telephone, and pager-based interactions with patients and nurses. Each of the patient cases and nursing actions are designed to heighten stress and simulate a “hot state” (System 1 thinking) environment by leveraging behavioral principles such as creating time pressure, increasing cognitive load, and reduced control (Table 1). Several irritants, including alerts from telemetry and intravenous pumps, also trigger throughout the cases to heighten stress, as literature suggests that noise annoyance is associated with increased anxiety [43,44]. Other behavioral strategies to induce stress and decisional conflict, such as action bias and social norming, are also incorporated in the scenario [45,46]. The scenario is also designed to help the physicians improve their communication skills with nurses, develop differential diagnoses, and consider alternative therapeutic options.

**Table 1:** Behavioral strategies implemented in simulation scenario and application to stressful prescribing situations.

Simulation behavioral strategy	Implementation in simulation scenario	Application to heightened stress and decisional conflict in high-risk prescribing
Time pressure	Repeated pages and demands by nursing for actively decompensating patients	Limited time is available to make treatment decisions; the need to be efficient enables affect heuristics and mental shortcuts
Cognitive load	Addressing and triaging 3 different patients and nurses with acute needs	Vast amounts of information must be processed to make prescribing decisions; the need to be efficient provokes System 1 thinking
Distraction/diverted attention	Telemetry beeping and patient intravenous pump alarms trigger loudly	These types of noises reduce the ability to easily process information and induce attentional bias
Reduced control	Clinically urgent patient with rapid ventricular response	Reduced ability to quickly respond to other patients enhances stress and urgency to prescribe quickly
Action bias	Nursing and patient demands for high-risk medication treatments	Tendency to favor action (eg, prescribing) over perceived inaction (eg, nonpharmacologic treatments), especially in stressful situations
Ambiguity effect	Nursing and patient demands for high-risk medication treatments and express displeasure with any alternatives	Clinical medicine curricula heavily focus on medications, priming interns to prescribe riskier medications
Social norming	Nursing pushback includes reference to what prior physicians have prescribed	Tendency to follow social “norms” presented by nurses and experiential training from peers enhance likelihood of poor prescribing

Prior to beginning each simulation, the facilitator briefly introduces the case and provides a shift-change handoff note with details of 5 hypothetical patients under their care, including brief summaries, tasks, and contingency plans. The simulation involves 3 patients set in the early evening. The intern is first called to the bedside by a nurse actor to address dyspnea for Patient 1, a 55-year-old man recently hospitalized for cellulitis and readmitted with pneumonia. Patient 1 develops atrial fibrillation with rapid ventricular response. While caring for Patient 1, a second nurse pages the intern several times about Patient 2, a 79-year-old man with mild cognitive impairment admitted with a wrist fracture after falling at home. The patient is agitated and disoriented. The nurse requests the intern to prescribe a sedating medication, despite its risks. When the intern goes to see Patient 2 in his hospital room, the simulated patient is attempting to get out of bed and yelling but is not physically violent. The patient is also attempting to remove his intravenous line and catheter.

As the scenario proceeds, another nurse pages twice about Patient 3, a 71-year-old woman admitted for an exacerbation of chronic obstructive pulmonary disease. She is distressed about having trouble sleeping and requesting a sleep medication. The nurse explains that the patient has already received melatonin without effect.

The intern will interact with the nurses for Patients 1 and 2 in person but will communicate with the nurse for Patient 3 only by telephone and pager, simulating the real-world distributed nature of care across units in hospital settings. If the intern tries to order nonpharmacological treatments for Patients 2 and 3, the nurses will also request that the intern prescribe antipsychotics, benzodiazepines, or sedative hypnotics, as applicable. If the intern refuses, the nurses will acquiesce initially but then continue to express concern. The scenario ends after the intern interacts twice with the nurse or patient, for all 3 patients.

#### Simulation Debriefing

Immediately after the scenario, the facilitator debriefs individually with each intern using a semistructured guide developed by the study team (Multimedia Appendix 3). The goal of the debriefing session is to reinforce the simulation session by discussing the decisional conflicts the intern experienced during the simulation. The facilitator asks each intern how they responded to stress and what choices they may have made if they had experienced less pressure. The debriefing also covers factors that led to increased stress, reasons for their prescribing decisions, alternative nonpharmacologic and pharmacologic options for managing insomnia and agitated delirium, and ways to improve communication with nursing staff and patients in future interactions. The debriefing lasts about 20 minutes for each intern.

### Control: Online Educational Training

Providers assigned to the control arm will receive self-directed online educational training about other treatments that are often overprescribed to hospitalized patients, specifically the transfusion of blood products, such as albumin and red blood cells [47,48]. Providing education about other high-risk medications will reduce nonspecific attention. This information is provided in the form of a 35-minute video lecture previously delivered several years prior by a local attending physician about transfusion reactions and management of blood products during a grand rounds talk. The lecture is housed on a website behind the hospital firewall (ie, not accessible outside of the hospital). After watching the video, the interns are asked to answer several clinical knowledge questions from the Biomedical Excellence for Safer Transfusion (BEST) Collaborative and American Society for Clinical Pathology’s validated physician knowledge examination and the study team [47]. We chose to use this mode of online training as lectures (and videotapes of the lectures) are commonly used during residency programs, and we wanted to evaluate the potential for spillover effects on other types of medications. As described above, both intervention and control arms were each designed to take about 40 minutes.

### Outcomes

The trial outcomes will be evaluated using structured electronic health record (EHR) data on the patient-level and a follow-up survey of the interns 2 weeks after they begin their evening rotation (Table 2). The trial’s primary outcome is the number of high-risk medication doses of antipsychotics, benzodiazepines, and sedative hypnotics prescribed per day to eligible patients (ie, ≥65 years of age, not on one of the medications upon admission) by the interns beginning on the day the trainings are delivered until the end of follow-up. We plan to exclude patients for the analysis who were on the relevant high-risk medication of interest prior to admission. For example, patients who were admitted on a benzodiazepine will not be included in the benzodiazepine/sedative hypnotic analysis but will be in the antipsychotic analysis.

**Table 2:** Study outcomes.

Outcome	Measurement	Assessment
Primary	High-risk medications prescribed per day: intern	Quantity of prescribed medication doses of high-risk medications (antipsychotics, benzodiazepines, sedative hypnotics) by the intern to patients ≥65 years not on treatment prior to admission over the follow-up period
Secondary	High-risk medications prescribed per day: all prescribers	Quantity of prescribed medication doses of high-risk medications (antipsychotics, benzodiazepines, sedative hypnotics) by all prescribers to patients ≥65 years not on treatment prior to admission over the follow-up period
Secondary	High-risk doses and types of medications prescribed per day	Strengths and types of medications of high-risk medications (antipsychotics, benzodiazepines, sedative hypnotics) by the intern to patients ≥65 years not on treatment prior to admission over the follow-up period
Secondary	Discharge medication order for high-risk medication: all prescribers	High-risk medication (antipsychotics, benzodiazepines, sedative hypnotics) prescribed to patients ≥65 years not on treatment prior to admission at hospital discharge
Secondary	Doses of spillover medications prescribed per day: intern	Quantity of prescribed medication doses for related medications (opioids, trazodone, melatonin) by the intern to patients ≥65 years not on treatment prior to admission over the follow-up period
Secondary	Doses of control medications prescribed per day: intern	Quantity of prescribed medication doses for control medications (eg, blood products) by the intern over the follow-up period

Given that patients have variable lengths of stay and, as a result, duration under the interns’ care, we will measure this outcome on the patient-day level to enable the fairest comparison between the arms. We will censor follow-up time on when patients transition from the intern’s service, including interns completing their evening service or death or hospital discharge. Medications ordered as needed will be treated the same as standing orders for the primary analysis. In secondary analyses, we will only measure doses that are standing orders and doses actually administered to patients. If patients are eligible to be measured for multiple medication classes (ie, antipsychotics and benzodiazepines or sedative hypnotics), we will sum the medication doses across the classes.

As secondary outcomes, we will measure: (1) the number of high-risk medication doses prescribed per eligible patient by all prescribers (ie, not only the enrolled intern) during the follow-up period; (2) the dose and type of high-risk medication doses prescribed during the follow-up period; (3) whether patients are ultimately discharged on 1 of these medications; (4) prescribing by the interns of other related medications such as opioids, trazodone, or melatonin, to measure spillover effects; and (5) rates of prescribing of blood products and albumin (eg, control medications to allow comparisons between the arms). We will also evaluate implementation outcomes informed by the Reach, Effectiveness, Adoption, Implementation, and Maintenance (RE-AIM) framework [49,50] from EHR and questionnaire data, including baseline characteristics of consenting and nonconsenting providers, whether the simulation training was completed, feedback and issues reported during the study, reported satisfaction with the intervention, and likelihood of incorporating insights into future practice. These other adoption and implementation outcomes will help us explore the extent to which the intervention could be used at scale.

### Analytic Plan and Sample Size Estimates

#### Analytic Plan

We will report means and frequencies of prerandomization variables in the intervention and control arms separately, comparing these values using absolute standardized differences.

To evaluate the primary outcome, we will use generalized estimating equations with a log-link function and Poisson-distributed errors, adjusting for patient- and physician-level clustering, and the block randomized design. We will include fixed effects for the treatment group and month of the year to account for seasonality. This approach will account for correlations between clustered observations. Because this is a randomized trial, our primary analyses are planned as unadjusted; however, if there are strong predictors of the outcomes not balanced by stratified randomization, we will adjust for these in the primary analyses. We will conduct analyses using intention-to-treat principles.

We will use a similar approach for the secondary outcomes. For discharge medication orders (secondary outcomes), we will also use generalized estimating equations that adjust for physician- and patient-level clustering and the block-randomized design using a logit-link function, binary-distributed errors, and fixed effects for the treatment group and month of the year. For the other secondary outcomes measuring prescribing by all prescribers, spillover effects, and prescribing of control medications, the approach will be the same, except using a log-link function and Poisson-distributed errors. Given the nature of the data, there will not be missing data for the primary outcome; however, there may be up to 25% of missingness for survey-based outcomes. Implementation outcomes, the STAI-6, and the Reactions to Uncertainty items will be descriptively compared at the provider level.

As secondary analyses, we plan to conduct subgroup analyses by provider sex, as prior work and the qualitative interviews with other medical resident physicians suggest a relationship between perceived authority on prescribing decisions and sex of the medical resident, which could affect high-risk prescribing decision making [51]. We will also explore differences by month of the year and inpatient service level, as System 1 thinking may be greater earlier in their training. In addition, we will also evaluate any time trends in prescribing outcomes over the follow-up period to explore whether the potential effect of being observed wanes over time.

#### Sample Size

We plan to recruit at least 36 interns for this trial (18 per arm), which should provide sufficient power to detect clinically meaningful differences in the primary outcome. Specifically, we estimated that we would have more than 80% power to detect a mean difference of 0.5 high-risk medication doses per patient-day in the intervention arm compared with the control arm, assuming an SD of 1.1, 2-sided α of .05, and intracluster correlations of 0.2 [21].

## Results

Recruitment of interns began on March 29, 2021. Recruitment for the trial ended in December 2021 with 40 total interns. Follow-up for study outcomes finished in January 2022. As of January 2022, we have begun data extraction and statistical analysis.

## Discussion

While gaps in System 1 and System 2 thinking are thought to contribute to decisional conflict in prescribing, this behavioral principle has not, to our knowledge, been explicitly addressed as part of a simulation intervention [1]. Accordingly, we launched a pilot trial evaluating the impact of a simulation-based training program designed to address decisional gaps between System 1– and System 2–driven choices on high-risk prescribing by medical resident physicians at a large academic medical center.

Simulation has increasingly been used to help health care professionals, alone and in teams, practice how they would handle stressful situations such as cardiac arrest or traumas in emergency rooms [25,27,28,30,31,52-54]. By extension, leveraging behavioral science principles within simulation to address decisional gaps between System 1– and System 2–driven choices holds great promise for reducing prescribing of high-risk medications for older adults. Prior interventions to specifically reduce the use of high-risk medications may only have been modestly successful because they have typically focused on educating providers and may not have adequately prepared them for making complex and urgent therapeutic decisions [1,22].

Further, despite growing knowledge and an evidence base for simulation-based interventions for providers and health care professionals, most existing studies on simulation have often evaluated changes in “cold-state” outcomes such as self-reported knowledge, changes in attitudes, or prescribing for simulated patients rather than clinical outcomes of patients in real-world practice [25-27,31]. Conversely, we are leveraging data directly from EHRs about participants’ actual prescribing to evaluate real-world clinical outcomes of simulation-based interventions.

There are several limitations that should be acknowledged. First, owing to the nature of the residency schedule, the length of follow-up for outcomes will be limited, and it will not be possible to evaluate long-term durability of outcomes. Second, while we are using expert facilitators and trained actors for the simulation sessions, there may be some variability in the actual scenarios based on intern prescribing decisions, as with any simulation. Third, it is possible that some prescribing decisions during follow-up in the real-world may not be fully the intern’s choice, but the intern is largely responsible for the care of patients during evening shifts, and we do not expect this or pressure by others on the care team, such as nurses, to be differential between arms. Similarly, the interns may prescribe slightly differently owing to their knowing they are participating in a trial, but we will explore the extent to which this occurs using a sensitivity analysis. Fourth, the content of the high-risk medications training differed between the arms, but we are measuring and comparing prescribing to both types of high-risk medications as outcomes. Fifth, while the interventions in both arms are only accessible to interns assigned to those arms, there is a hypothetical risk of contamination, but this would bias results toward the null. Sixth, System 1 thinking may be efficient in some clinical settings, so addressing this principle may be insufficient to optimize prescribing [55-57]. Finally, these findings may not generalize to medical residents in nontertiary health care systems or more senior physicians.

In conclusion, this trial will evaluate the impact of a simulation-based training program designed using behavioral science on prescribing of high-risk medications by junior physicians. If the intervention is shown to be effective, this approach is expected to be reproducible in other clinical environments and for a broader set of behaviors. Regardless of outcome, the trial will also provide additional insight into the real-world effectiveness of simulation-based training and help tailor evidence-based medicine education.

## Appendix

Multimedia Appendix 1 6-item State-Trait Anxiety Inventory (STAI-6).

Multimedia Appendix 2 The Revised Physicians' Reactions to Uncertainty Scales.

Multimedia Appendix 3 Debriefing guide.

## Abbreviations

BEST: Biomedical Excellence for Safer Transfusion
BWH: Brigham and Women’s Hospital
EHR: electronic health record
RE-AIM: Reach, Effectiveness, Adoption, Implementation, and Maintenance
STAI-6: 6-item State-Trait Anxiety Inventory
