# Supplementary material for: Overcoming Decisional Gaps in High-Risk Prescribing by Junior Physicians Using Simulation-Based Training: Protocol for a Randomized Controlled Trial
Source: JMIR Res Protoc. 2022 Apr 27;11(4):e31464. doi: 10.2196/31464 (PMC9096643; doi:10.2196/31464)
Supplement: Multimedia Appendix 1 [file resprot_v11i4e31464_app1.docx]

| Read each statement and select the appropriate response to indicate how you feel when making stressful prescribing decisions for patients. There are no right or wrong answers. Do not spend too much time on any one statement but give the answer which seems to describe your present feelings best. | | | | |
| --- | --- | --- | --- | --- |
| 1. I feel calm | 1  Not at all | 2  A little | 3  Somewhat | 4  Very Much So |
| 2. I feel tense | 1  Not at all | 2  A little | 3  Somewhat | 4  Very Much So |
| 3. I feel upset | 1  Not at all | 2  A little | 3  Somewhat | 4  Very Much So |
| 4. I am relaxed | 1  Not at all | 2  A little | 3  Somewhat | 4  Very Much So |
| 5. I feel content | 1  Not at all | 2  A little | 3  Somewhat | 4  Very Much So |
| 6. I am worried | 1  Not at all | 2  A little | 3  Somewhat | 4  Very Much So |
