# Supplementary material for: Overcoming Decisional Gaps in High-Risk Prescribing by Junior Physicians Using Simulation-Based Training: Protocol for a Randomized Controlled Trial
Source: JMIR Res Protoc. 2022 Apr 27;11(4):e31464. doi: 10.2196/31464 (PMC9096643; doi:10.2196/31464)
Supplement: Multimedia Appendix 2 [file resprot_v11i4e31464_app2.docx]

| Read each statement and select the appropriate response to indicate how you feel when making stressful prescribing decisions for patients. There are no right or wrong answers. Do not spend too much time on any one statement but give the answer which seems to describe your present feelings best. | | | | | | |
| --- | --- | --- | --- | --- | --- | --- |
| Anxiety due to Uncertainty (5 items) | | | | | | |
| 1. I usually feel anxious when I am not sure of a diagnosis. | 1 Strongly Disagree | 2 Moderately Disagree | 3 Slightly Disagree | 4 Slightly Agree | 5 Moderately Agree | 6 Strongly Agree |
| 1. I find the uncertainty involved in patient care disconcerting. | 1 Strongly Disagree | 2 Moderately Disagree | 3 Slightly Disagree | 4 Slightly Agree | 5 Moderately Agree | 6 Strongly Agree |
| 1. Uncertainty in patient care makes me uneasy. | 1 Strongly Disagree | 2 Moderately Disagree | 3 Slightly Disagree | 4 Slightly Agree | 5 Moderately Agree | 6 Strongly Agree |
| 1. I am quite comfortable with the uncertainty in patient care. | 1 Strongly Disagree | 2 Moderately Disagree | 3 Slightly Disagree | 4 Slightly Agree | 5 Moderately Agree | 6 Strongly Agree |
| 1. The uncertainty of patient care often troubles me. | 1 Strongly Disagree | 2 Moderately Disagree | 3 Slightly Disagree | 4 Slightly Agree | 5 Moderately Agree | 6 Strongly Agree |
| Concern About Bad Outcomes (3 items) | | | | | | |
| 1. When I am uncertain of a diagnosis, I imagine all sorts of bad scenarios-- patient dies, patient sues, etc | 1 Strongly Disagree | 2 Moderately Disagree | 3 Slightly Disagree | 4 Slightly Agree | 5 Moderately Agree | 6 Strongly Agree |
| 1. I fear being held accountable for the limits of my knowledge. | 1 Strongly Disagree | 2 Moderately Disagree | 3 Slightly Disagree | 4 Slightly Agree | 5 Moderately Agree | 6 Strongly Agree |
| 1. I worry about malpractice when I do not know a patient's diagnosis. | 1 Strongly Disagree | 2 Moderately Disagree | 3 Slightly Disagree | 4 Slightly Agree | 5 Moderately Agree | 6 Strongly Agree |
