# Supplementary material for: Overcoming Decisional Gaps in High-Risk Prescribing by Junior Physicians Using Simulation-Based Training: Protocol for a Randomized Controlled Trial
Source: JMIR Res Protoc. 2022 Apr 27;11(4):e31464. doi: 10.2196/31464 (PMC9096643; doi:10.2196/31464)
Supplement: Multimedia Appendix 3 [file resprot_v11i4e31464_app3.docx]

| 1. Allow for an emotional release – “How did that feel?” or “How do you think that went?”    1. How much stress did it feel like you were under?    2. When was the last time you cared for a patient like this and how did you feel? |
| --- |
| 1. Discuss non-pharmacologic options for managing agitated delirium as a first step    1. Overview of etiologies, also medication-related (e.g., anticholinergic medications), indwelling catheters       1. Withdrawal of maintenance meds stopped on admission?    2. What non-pharmacologic options did you consider, and why did you choose XXX?       1. Redirection, bed alarm, frequent checks, SAFE responses (participate, less likely to get called later)    3. Tips for communicating rationale for non-pharm management to nurses and patients |
| 1. Prioritizing non-sedative/non-benzo medications for agitated delirium    1. Role (or rather non-role) of using the EKG/QTc for guiding choice of treatment       1. What crossed your mind when you received the EKG?          1. Non-elevated QTc does not mean that there may not be a problem in prescribing       2. May often have delayed QTc or unable to get (e.g., too agitated to put on leads)       3. QT-prolonging conditions (e.g., electrolyte abnormalities, other drugs, hypothyroid)    2. Roles of Haldol, trazodone, Depakote, Seroquel       1. Avoid all if possible but PO first line, lowest possible dose          1. Quetiapine 12.5mg PO best possible choice if need medication             1. Second line: olanzapine 2.5mg PO          2. Some guidelines: Haldol small dose (but less recommended) |
| 1. Discuss non-pharmacologic and first-line options for managing insomnia    1. What crossed your mind when you were considering what to prescribe to Patient 3?       1. Modifying lab draws/cluster care       2. Melatonin       3. Tips for communicating rationale for non-pharm management to nurses and patients |
| 1. Avoiding prescribing of benzos/Z-drugs for either condition, especially for insomnia    1. Discuss risks, even short-term use    2. Avoid in elderly unless alcohol withdrawal |
| 1. When to worry – emphasis on specific reasons to escalate (to resident, attending, psych, security)    1. Failing to respond    2. Actual physical harm to self, nurse, or other patients |
| 1. Have you been in a situation like this and how did you feel?    1. How does this compare and contrast with the simulation scenario you just experienced?    2. Many people have been in anxiety-provoking situation on the wards. Given what we talked about today, are there things you would do differently? |
| 1. What is one thing you’ll take with you back to your twilight rotation? |
